# Novel nutraceutical combination restores hepatic deiodinase expression and protein levels under inflammatory conditions: evidence from an *in vitro* model

**DOI:** 10.3389/fnut.2026.1773486

**Published:** 2026-03-26

**Authors:** E. Valeri, F. Loupakis, G. Pasqualetti, E. Paccosi, L. Proietti De Santis, S. Filippi

**Affiliations:** 1 Unit of Molecular Genetics of Aging, Department of Ecological and Biological Sciences, University of Tuscia, Viterbo, Italy; 2 3Trees Healthcare, Viterbo, Italy; 3 Kiss Association ETS, Pisa, Italy

**Keywords:** deiodinase, nutraceutical combination, FT3/FT4 ratio, malnutrition, nutritional supplement, sarcopenia

## Abstract

**Background/objectives:**

Thyroid hormone activation depends on the conversion of T4 to T3 by the selenoenzyme DIO1, whose expression is suppressed by inflammation and oxidative stress. This study evaluated whether a combination of vitamin A, selenium, taurine, oleic acid, and resveratrol could counteract Lipopolysaccaride (LPS)-induced downregulation of DIO1 in HepG2 cells.

**Methods:**

Cytotoxicity of Taurine, Resveratrol, Retinol, and Oleic acid was assessed in FB789 and HepG2 cells by MTT assay. Non-toxic, biologically active concentrations (2.5 μM Taurine; 2.5 μM Resveratrol; 50 μM Retinol; 100 μM Oleic Acid) were used to test DIO1 modulation following LPS exposure (1 μg/mL). *DIO1* mRNA and protein levels were quantified by qRT-PCR and Western blot.

**Results:**

All compounds exhibited acceptable cell viability profiles at low-to-mid range doses. LPS markedly suppressed *DIO1* mRNA expression, whereas each nutrient partially restored its levels. Notably, the combined treatment completely prevented LPS-mediated DIO1 downregulation and significantly increased DIO1 protein abundance compared with both medium and LPS-treated controls.

**Conclusion:**

In this *in vitro* model, a specific combination of bioactive nutrients effectively restored DIO1 expression under inflammatory conditions, supporting peripheral thyroid hormone activation. These findings provide mechanistic rationale for nutrition-based strategies aimed at mitigating inflammation-related impairment of T4-to-T3 conversion in vulnerable or catabolic clinical populations.

## 1 Introduction

Thyroid hormones are key regulators of energy metabolism, redox homeostasis, and protein turnover in multiple tissues. Their biological activity largely depends on the local and systemic conversion of thyroxine (T4) to triiodothyronine (T3), a reaction catalyzed by the selenoprotein iodothyronine deiodinase type 1 (DIO1). This enzyme is highly expressed in the liver and kidney and plays a central role in maintaining circulating T3 concentrations and in modulating metabolic adaptation to stress (1, 2).

DIO1 activity is tightly regulated by nutritional factors, cytokines, and oxidative stress. Several *in vitro* and *in vivo* studies have shown that exposure to lipopolysaccharide (LPS) or inflammatory mediators such as interleukin-6 and tumor necrosis factor-*α* suppresses *DIO1* gene expression, leading to a decrease in peripheral T3 production (3–5). This mechanism is considered an adaptive metabolic response aimed at reducing energy expenditure during acute stress; however, when persistent, it may contribute to tissue dysfunction, loss of muscle mass, and adverse clinical outcomes (6, 7).

Recent evidence highlights that the ratio between free triiodothyronine (fT3) and free thyroxine (fT4) represents a sensitive biomarker of peripheral deiodination efficiency and overall metabolic resilience. A low fT3/fT4 ratio has been associated with systemic inflammation (8), frailty (9, 10), sarcopenia (11), malnutrition (12), and unfavorable clinical outcomes in both medical and oncologic settings (13–18). Collectively, these data suggest that the preservation of adequate T4-to-T3 conversion capacity may reflect a fundamental determinant of metabolic health, linking nutritional status, inflammation, and endocrine adaptation.

Inflammation-induced DIO1 downregulation is mediated by the activation of NF-κB–dependent pathways and by the accumulation of reactive oxygen and nitrogen species that alter selenoprotein synthesis and function (19). In experimental models, the suppression of DIO1 by LPS or cytokines can be attenuated by antioxidants or anti-inflammatory agents (20), supporting the hypothesis that nutritional and redox status modulate thyroid hormone metabolism at the cellular level. Selenium, a structural component of DIO1, is indispensable for its catalytic activity, while other nutrients such as Retinoids, Taurine, Monounsaturated fatty acids, and polyphenols (e.g., Resveratrol) exert complementary actions on transcriptional effects, oxidative balance, mitochondrial biogenesis, and cytokine signaling (21).

From a mechanistic perspective, these compounds may help preserve DIO1 expression and maintain physiological T3 availability in conditions of inflammatory stress. The interplay between nutrient signaling and thyroid hormone metabolism therefore represents a promising area for nutritional intervention, particularly in populations at risk of chronic inflammation, catabolic diseases, or age-related functional decline.

Based on these considerations, the present study aimed to explore, in an *in vitro* model of LPS-induced inflammation, the effects of a combination of selected nutrients—vitamin A, selenium, taurine, oleic acid, and resveratrol—on the expression and activity of DIO1. The objective was to determine whether these bioactive compounds could mitigate inflammation-induced suppression of peripheral thyroid hormone activation, thereby supporting a more favorable redox and metabolic profile.

## 2 Materials and methods

### 2.1 Cell lines

FB789, a human normal fibroblast and Hepatoma cancer (HEPG2) cell lines were used to assess both the cytoxicity and *DIO1* gene modulation after nutraceutical compounds treatment.

FB789 and HEPG2 cells were cultured according to Filippi et al. (22) and Berni et al. (23).

### 2.2 Evaluation of cell viability after nutraceuticals and lipopolysaccharide (LPS)

Cells were seeded 18 h before treatment in a concentration of 3×10^3^ for each experimental point in a 96 well/plate.

Taurine and Retinol were used in a range of 2.5 to 100 μM, Resveratrol was used in a range of 2.5 to 200 μM, while Oleic Acid was used at 100 μM. LPS (1 μg/mL) was added 1 h before nutraceutical compounds. Selenium was not supplemented in the culture medium, as fetal bovine serum (FBS) is generally considered to provide sufficient selenium levels. Specifically, the FBS used in our experiments contains a selenium concentration of 15.9 ug/L.

Cell viability was evaluated by measuring the mitochondrial-dependent conversion of the yellow tetrazolium salt MTT [3-(4.5-dimethylthiazol-2-yl)-2.5-diphenyl-2H-tetrazolium bromide] to a purple formazan crystal by metabolically active cells. The experiments were conducted in triplicate according to Botta et al. (24).

### 2.3 Gene expression analyses after nutraceuticals and LPS treatment

1×10^5^ were seeded in a 60 mm dish 18 h before treatment. The concentrations of the nutraceutical compounds used in the experiments were derived from both cytotoxicity dose–response curves and DIO-1 mRNA levels performed for each compound. Based on these analyses we selected the lowest concentrations of each compound (including resveratrol, taurine, and retinol) that are reported to exert biological activity, in order to avoid cytotoxic effects that could interfere with the interpretation of the results. RNA was isolated using NucleoSpin RNA kit (Macherey-Nagel, GmBH & CO., Dueren, Germany) according to the manufacturer’s instructions. RNA was diluted by adding 40 μL of DEPC water and its integrity was checked on a denaturing 1% agarose gel. RNA concentration was measured with Qubit Fluorometer 2.0 cDNA synthesis, which was performed with 1 μg of RNA for each sample by using the First Strand cDNA Synthesis kit (Thermo Fisher Scientific, Waltham, MA, United States). Comparative qRT-PCR was carried out with GoTaq qPCR SYBR green master mix (Promega, Madison, WI, United States), using Mx3005P Real-Time PCR system (Agilent, Santa Clara, CA, United States). Results were normalized to *β*-Actin. Primer sequences are available on request.

### 2.4 Protein expression analysis by Western blot

Proteins from HEPG2 cell line was fractionated by SDS-PAGE and transferred to nitrocellulose membrane (Biorad Laboratories, Hercules, CA, United States), according to Botta et al. (25). After blotting, the membrane was incubated with appropriate primary and secondary antibodies. Primary antibodies used were against DIO-1 (Immunological Science) and *β*-Actin (Santa Cruz Biotecnology, Santa Cruz, CA, United States), while secondary antibodies were HRP conjugated.

### 2.5 Statistical analysis

Data are presented as mean ± standard deviation (SD) from at least three independent experiments. Statistical analyses were performed using GraphPad Prism 9 (GraphPad Software, San Diego, CA, United States). Comparisons between multiple groups were conducted using one-way analysis of variance (ANOVA), followed by Turkey’s multiple comparisons test to correct for multiple comparisons. A *p*-value < 0.05 was considered statistically significant.

## 3 Results

### 3.1 Cytotoxicity analysis after nutraceuticals treatment

**Figure 1:**
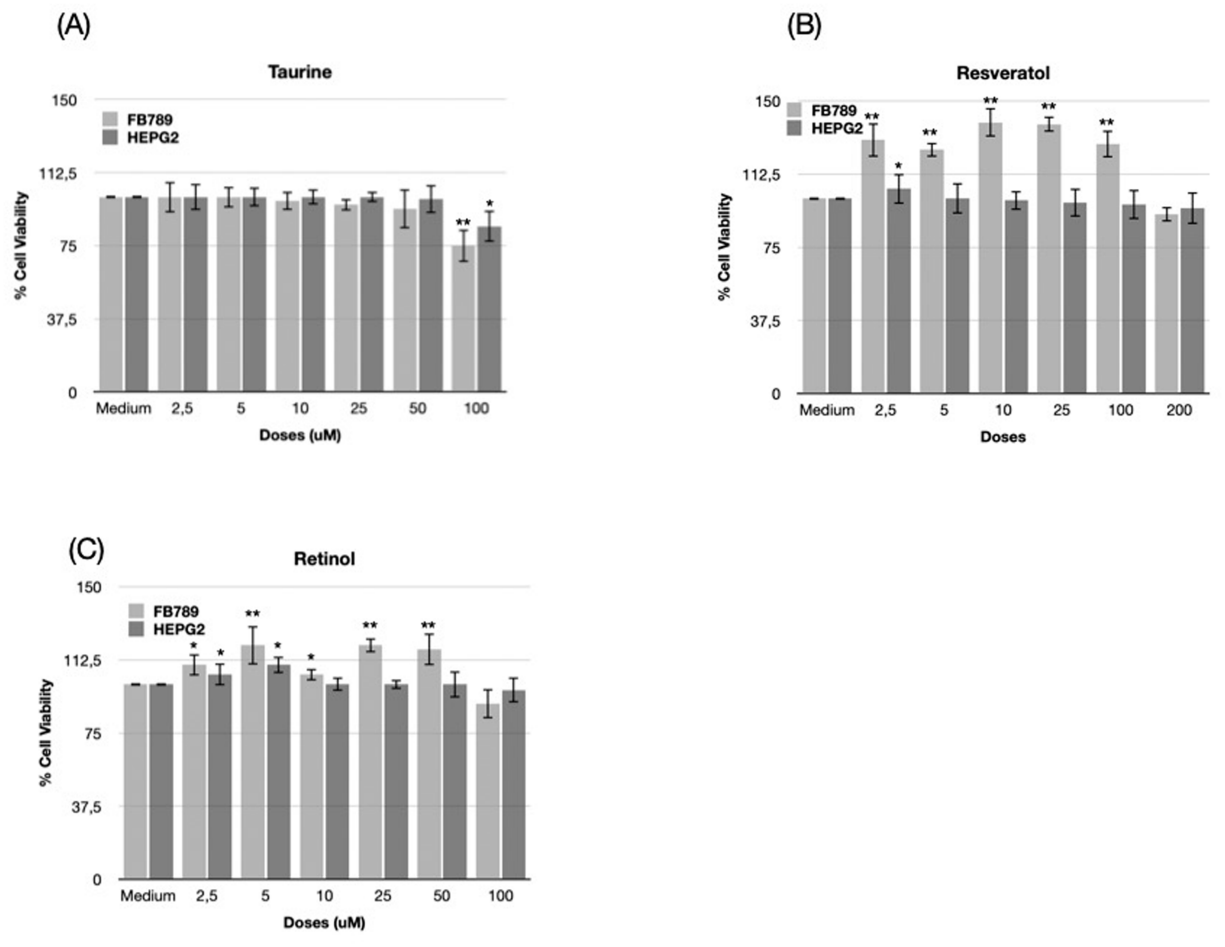
Bar presents the percentage effects of different nutraceutical concentrations on the viability of two cell lines, FB789 and HEPG2 for taurine, resveratrol, and retinol, respectively **(A–C)**. Data for each condition are shown as mean ± standard deviation (SD). Error bars indicate the standard deviation of replicates, highlighting the reproducibility and variability within each condition (*p < 0.05 **p < 0.01, medium vs. treatment).

Cell viability was measured as a percentage relative to the untreated control (Medium). Figure 1 presents the effects of different nutraceutical concentrations on the viability of two cell lines, FB789 and HEPG2.

The graph (panel A) shows the effect of increasing concentrations of Taurine (2.5, 5, 10, 25, 50, and 100 μM) on the cell viability of FB789 and HEPG2 cell lines, compared to untreated control (medium). Cell viability remains close to control levels for both cell lines up to 25 μM taurine. At higher concentrations (50 and 100 μM), a reduction in cell viability becomes evident, particularly at 100 μM, where both FB789 and HEPG2 show a notable, but not drastic, decrease in viability. FB789 cells appear slightly more sensitive at the highest doses compared to HEPG2 (25–29).

Panel B shows the resveratrol concentrations tested (μM) were 2.5, 5, 10, 25, 100, and 200. Upon exposure to the compound: FB789 cells exhibited a dose-dependent increase in viability at lower concentrations (2.5–25 μM), peaking at 10 μM. A gradual decrease was observed at higher concentrations (100 and 200 μM), though viability remained elevated compared to the control. HEPG2 cells showed a different profile. After an initial slight increase at 2.5 μM, viability decreased steadily with higher doses, reaching the lowest values at 200 μM.

The graph (panel C) illustrates the impact of increasing concentrations of retinol (2.5, 5, 10, 25, 50, and 100 μM) on the viability of FB789 and HEPG2 cell lines, compared to untreated controls. In both cell lines, cell viability increases moderately at low-to-intermediate doses (peaking notably at 5 and 25 μM, more pronounced in FB789), followed by a slight reduction at the highest concentrations (50 and 100 μM).

### 3.2 Modulation of both mRNA levels and protein expression of DIO-1 after nutraceutical treatment

Based on the cytotoxicity results and dose response single compound DIO-1 inductions, we selected a single dose for each nutraceutical compound, specifically 2.5 μM for both Taurine and Resveratol, 50 μM for Retinol for the modulation of DIO-1 after concurrent nutraceutical compound treatments. Higher concentrations did not result in a further increase in DIO1; on the contrary, we observed a decrease, likely related to toxicity.

The dose of oleic acid (OA) and lipopolysaccharide (LPS) was chosen on the basis of the literature and corresponds to 100 μM and 1 μg/mL, respectively (30, 31).

The results demonstrate differential regulation of DIO-1 expression at the mRNAlevels in HEPG2 cells under various treatments.

**Figure 2:**
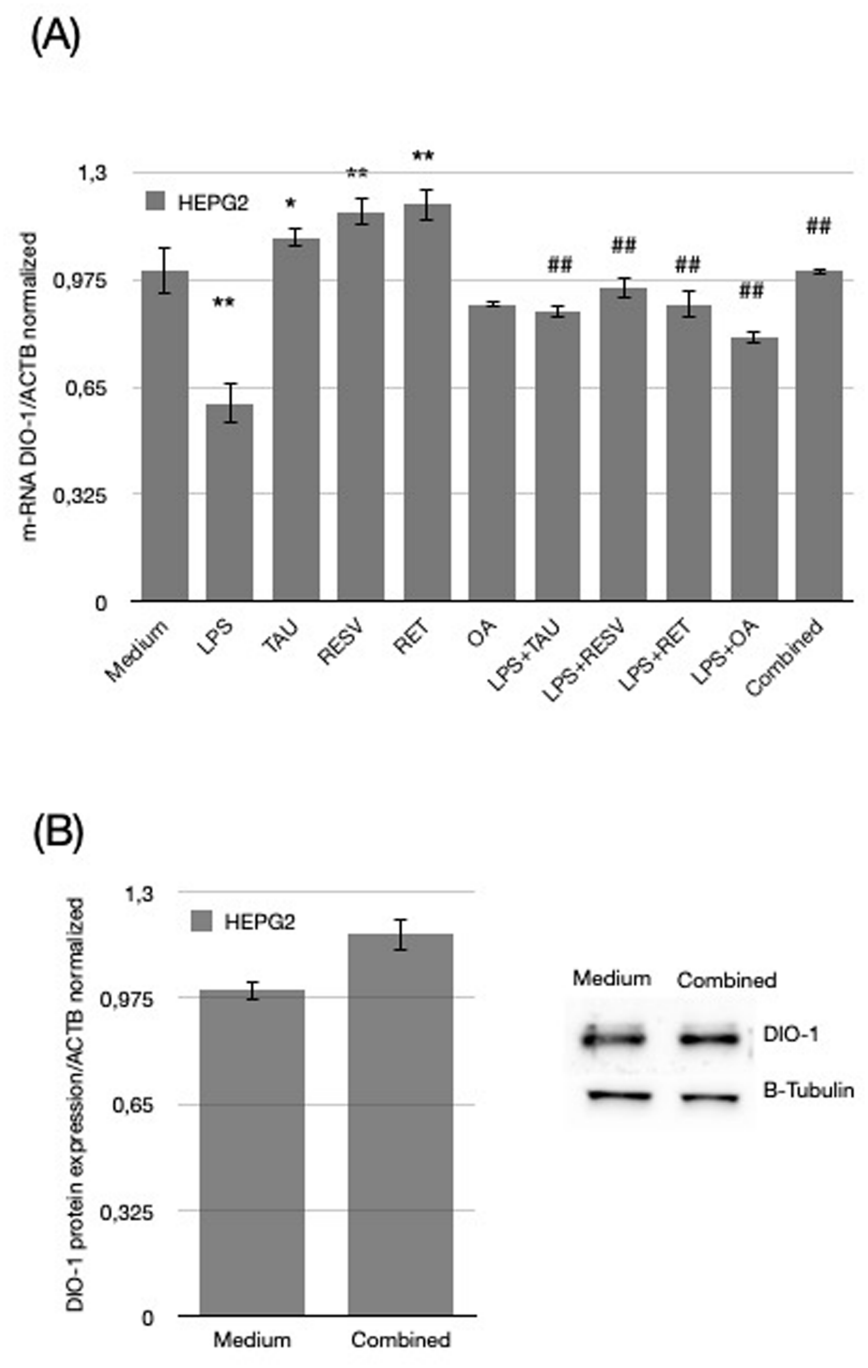
**(A)** Levels of *DIO-1* mRNA expression after HEPG2 cells were treated with specific doses of nutraceutical compounds; last bar refers to the simultaneous treatment with all the compounds. **(B)** Shows DIO-1 protein levels when cells were treated at the same time with both LPS and the combined nutraceutical compounds (OA, RET, RESV, TAU), compared to control. Data for each condition are shown as mean ± standard deviation. Error bars represent the standard deviations of replicates, highlighting the reproducibility and variability within each condition (*p < 0.05, Medium vs. LPS or Medium vs. Combined; #*p* < 0.05, ##*p* < 0.01, LPS vs. LPS + RESV or LPS vs. Combines).

In detail, LPS exposure significantly caused a pronounced down-regulation of *DIO-1* mRNA expression *actin* normalized (0.6 ± 0.03, *p* < 0.01), whereas taurine, resveratrol, and retinol increased DIO-1 mRNA levels (1.1 ± 0.2, 1.18 ± 0.03, 1.2 ± 0.07 respectively). Oleic acid (OA) did not affect DIO-1 expression (0.9 ± 0.05) (Figure 2).

When cells were exposed to LPS in combination with each single agent, *DIO-1* mRNA levels were increased compared with LPS-treated cells; however, none of the treatments completely restored the expression to baseline *actin* normalized levels (0.88 ± 0.03 for LPS + TAU, 0.95 ± 0.07 for LPS + RESV, 0.9 ± 0.02 for LPS + RET and 0.8 ± 0.05 for LPS + OA). Only the combined treatment with all four elements markedly increased DIO-1 expression compared with LPS reaching the control *actin* normalized levels (1 ± 0.09 for Combined, *p* < 0.01) as shown in Figure 2.

Figure 2B presents data on DIO-1 protein expression in HEPG2 cells following a simultaneous treatment. The “Combined” treatment markedly increased DIO-1 protein expression *actin* normalized (1.17 ± 0.05 for combined, *p* < 0.01) in cells simultaneously exposed to LPS compared to medium control.

## 4 Discussion

This *in vitro* study demonstrates that DIO1 expression in hepatic cells is markedly downregulated in response to lipopolysaccharide (LPS)–induced inflammation and treatment with a combination of taurine, resveratrol, retinoic acid, and oleic acid effectively restored DIO1 mRNA expression. Moreover, at the protein level, the combined treatment markedly increased DIO-1 expression even in the presence of LPS compared with the medium control, indicating a synergistic or cumulative effect of the combined factors in modulating DIO-1 abundance. Cytotoxicity assays confirmed the absence of significant toxicity in both cell lines across the tested concentration range, with cell viability remaining above 75% in all conditions (32).

We selected the concentrations of resveratrol, taurine, and retinol for DIO1 modulation assays by combining cytotoxicity data with single-compound dose–response experiments, in order to identify the most biologically effective but non-toxic range. Notably, the observed DIO1 modulation occurred at concentrations well below the cytotoxic threshold, supporting the physiological relevance of the findings. Previous *in vitro* studies used resveratrol concentrations between 0.5 and 5 μM to achieve biological activity (33), and the mean serum concentration after a 500 mg oral dose in healthy volunteers is about 0.3 μM (34). Plasma retinol concentrations after 20 mg supplementation reach 1.3 μM (35), while normal taurine plasma levels are around 40 μM-approximately 15-fold higher than the concentration used in our experiments. For oleic acid, typical plasma concentrations range from 0.2 to 5.0 mmol/L (36) and the 100 μM concentration applied here is comparable to postprandial levels following a MUFA meal. We selected a relatively low MUFA concentration to allow the detection of potential interactions with the other compounds, while avoiding a dominant effect of MUFA alone, which at higher concentrations can increase DIO1 expression by 3–4-fold (37).

In this experiment, taurine, resveratrol, and retinoic acid individually increased DIO1 mRNA levels compared with the control, in line with their antioxidant properties and transcriptional effects on hepatic metabolism. At the molecular level, LPS exposure significantly reduced DIO1 transcript abundance, in line with prior evidence showing that inflammatory cytokines and oxidative stress suppress deiodinase activity in hepatic and other systems (3, 19). DIO1 downregulation under inflammatory conditions is likely mediated by activation of the NF-κB and MAPK (p38/JNK) pathways, which are known to suppress thyroid hormone–metabolizing enzymes (4). The nutraceuticals tested may counteract inflammatory suppression of DIO1 through their anti-inflammatory actions: retinol and resveratrol inhibit NF-κB signaling, with resveratrol also modulating MAPK activity; taurine reduces NF-κB activation and ROS-dependent MAPK signaling; and oleic acid attenuates TLR4-mediated inflammatory pathways (38–41).

Importantly, concurrent treatment with all four nutrients fully counteracted the LPS-induced suppression of DIO1 expression, suggesting a synergistic or complementary mechanism that stabilizes thyroid hormone activation under inflammatory stress. The partial discrepancy between mRNA and protein expression may reflect post-transcriptional regulation or differences in protein stability (2). Mechanistically, these nutrients exert converging antioxidant and anti-inflammatory effects—taurine and oleic acid modulate oxidative tone and cytokine signaling, resveratrol enhances transcriptional effects and mitochondrial biogenesis, and retinoic acid acts via nuclear receptors—while selenium, although not directly tested, is indispensable for DIO1 catalytic activity.

These findings support the concept that inflammation-driven suppression of DIO1 contributes to impaired fT4-to-fT3 conversion, a frequent event in chronic inflammatory and catabolic states (12, 36). Persistent reduction in peripheral T3 generation correlates with frailty, sarcopenia, and poor clinical outcomes across various conditions (9–11, 13–18). The present study thus provides mechanistic insight into how specific nutrients may counteract this impairment, offering biological plausibility for nutritional strategies that sustain thyroid hormone activation.

From a practical perspective, nutritional supplements or food for special medical purposes (FSMPs) enriched with bioactive compounds such as taurine, resveratrol, vitamin A, oleic acid, and selenium could represent innovative tools to address the complex malnutrition frequently observed in older, pre-frail, and frail adults, as well as in cancer patients. In these settings, low-grade systemic inflammation, oxidative stress, and nutrient deficiencies often converge to blunt fT4-to-fT3 conversion, leading to reduced metabolic efficiency, muscle wasting, and poorer tolerance to therapy (9–12, 14–18). By combining antioxidant and anti-inflammatory nutrients capable of supporting deiodinase activity, next-generation FSMPs could provide a new nutritional strategy to preserve muscle integrity, enhance treatment tolerance, and improve outcomes in these high-risk populations.

Similar alterations in thyroid hormone activation have been described in other chronic disorders, including inflammatory bowel disease, Parkinson’s disease, dialysis-dependent renal failure, rheumatoid arthritis, and anorexia nervosa, suggesting that deiodinase modulation may be a unifying nutritional target across multiple inflammatory and catabolic diseases (42–45).

A limitation of this study is the use of a single cellular model, which cannot replicate systemic thyroid hormone regulation or tissue cross-talk. Moreover, this is an *in vitro* model and it may not be fully predictive of *in vivo* responses to specific compounds, given the complexity of kinetic factors and systemic interactions. Nevertheless, the consistency between these results and clinical observations supports further research into nutritional interventions designed to preserve fT4-to-fT3 conversion and metabolic homeostasis under inflammatory stress. In the present study, our experiments focused only on *DIO1* gene expression without a proper enzyme activity assay. The relationship between gene expression or protein abundance of selenoproteins and their overall enzymatic activity is not strictly linear and depends on several factors such as selenium availability. In these experiments, selenium was not supplemented in the culture medium because it was already present at a concentration comparable to that found in human serum (46). Finally, the stability of the bioactive compounds in the culture medium was not directly measured. However, since the treatment duration was relatively short (24 h), we assume that the compound concentrations remained sufficiently stable throughout the experiment.

In conclusion, concurrent exposure to taurine, resveratrol, oleic acid, and vitamin A effectively prevented inflammation-induced DIO1 suppression. These findings reinforce the hypothesis that targeted nutritional modulation can mitigate inflammation-related impairment of fT4-to-fT3 conversion, offering a promising avenue for maintaining endocrine and metabolic health in frail, elderly, or oncologic patients and potentially across a broader spectrum of chronic inflammatory conditions.

## Data Availability

The original contributions presented in the study are included in the article. Further inquiries can be directed to the corresponding author.
